# Epigenetic Landscape of DNA Methylation in Pancreatic Ductal Adenocarcinoma

**DOI:** 10.3390/epigenomes8040041

**Published:** 2024-11-03

**Authors:** Peiyi Liu, Juliette Jacques, Chang-Il Hwang

**Affiliations:** 1 Department of Microbiology and Molecular Genetics, College of Biological Sciences, University of California, Davis, Davis, CA 95616, USA; pyiliu@ucdavis.edu (P.L.); jrjacques@ucdavis.edu (J.J.); 2 University of California Davis Comprehensive Cancer Center, University of California, Davis, Sacramento, CA 95817, USA

**Keywords:** DNA methylation, pancreatic ductal adenocarcinoma, pancreatic cancer, whole-genome bisulfite sequencing, metastasis

## Abstract

Pancreatic ductal adenocarcinoma (PDAC) remains one of the most lethal malignancies, characterized by its aggressive progression and dismal prognosis. Advances in epigenetic profiling, specifically DNA methylation analysis, have significantly deepened our understanding of PDAC pathogenesis. This review synthesizes findings from recent genome-wide DNA methylation studies, which have delineated a complex DNA methylation landscape differentiating between normal and cancerous pancreatic tissues, as well as across various stages and molecular subtypes of PDAC. These studies identified specific differentially methylated regions (DMRs) that not only enhance our grasp of the epigenetic drivers of PDAC but also offer potential biomarkers for early diagnosis and prognosis, enabling the customization of therapeutic approaches. The review further explores how DNA methylation profiling could facilitate the development of subtype-tailored therapies, potentially improving treatment outcomes based on precise molecular characterizations. Overall, leveraging DNA methylation alterations as functional biomarkers holds promise for advancing our understanding of disease progression and refining PDAC management strategies, which could lead to improved patient outcomes and a deeper comprehension of the disease’s underlying biological mechanisms.

## 1. Introduction

Pancreatic cancer is the third leading cause of cancer-related deaths in the United States and is projected to take the place of colorectal cancer as the second leading cause by 2030 [1,2]. It is relatively uncommon but exceptionally lethal, characterized by the lowest five-year survival rate among common cancers at just 13% [1]. The most prevalent and most malignant form of pancreatic cancer is pancreatic ductal adenocarcinoma (PDAC), accounting for 90% of pancreatic cancer cases and resulting in nearly 50,000 deaths annually in the US alone [3]. Late diagnosis often due to asymptomatic early stages, frequent metastasis, and lack of effective treatment contributes to the poor survival outcomes of the disease. Less than 20% of patients are eligible for surgical resection of tumors, and even then, resistance to chemotherapy and radiotherapy, in part due to the characteristic dense stroma and desmoplastic reaction, limits the effectiveness of treatment [4]. Next-generation sequencing (NGS) has facilitated the discovery as well as the characterization of genetic aberrations that contribute to PDAC disease initiation and progression [5]. More specifically, 90–92% of PDAC patients have gain-of-function mutations in the *KRAS* proto-oncogene in exocrine pancreas cells. These oncogenic mutations lead to the initiation of disease and the formation of precancerous lesions known as pancreatic intraepithelial neoplasia (PanIN) [6,7,8]. As these lesions progress, inactivating mutations or deletions of classic tumor suppressor genes such as *CDKN2A*, *TP53*, and *SMAD4* further promote disease progression [5,9,10]. Despite a well-understood genetic mutational landscape, therapeutic applications remain limited, and understanding the mechanism of disease progression, namely metastasis, continues to pose a significant challenge.

Over the years, PDAC research has shifted to include the effects of epigenetic mechanisms on cancer progression [11,12,13,14]. It has become increasingly evident that epigenetic mechanisms play a role in disease pathology. Through their mechanism of transcription regulation, epigenetic alterations have profound effects on gene expression and genome stability and can equally promote oncogenic pathways. DNA methylation is one epigenetic mechanism that has widely been found dysregulated in many cancers, PDAC included [15,16,17,18]. DNA methylation predominately takes place at cytosines followed by guanines, or CpG regions. It is known to be negatively associated with gene expression at promoter and enhancer regions while being positively associated with gene expression at gene body regions, although developments in methylome analysis and further studies have suggested the relationship between methylation and gene expression is more complex than previously anticipated [19,20]. Advanced methods of investigating the methylome have enabled greater coverage of CpG sites and even non-CpG sites with higher resolution, allowing for more comprehensive studies on the role of DNA methylation to be conducted. However, further exploration is constrained by the accessibility and quality of the tumor samples. The dense stromal contents within the tumor microenvironment lead to stromal interference, posing the problem of low cellularity when attempting to isolate neoplastic epithelial cells for investigating their molecular mechanisms. These epithelial cells are the minority among the large diversity of cell types that comprise the surrounding stroma, complicating the isolation of pure neoplastic populations. As a result, extensive efforts are being made toward understanding disease characterization and progression, along with the development of original techniques to effectively isolate tumor cells.

In this review, we will discuss the current status of studies investigating the role of DNA methylation in PDAC progression [14]. We will equally highlight two recently published comprehensive DNA methylome studies in PDAC patients, each using unique techniques to circumvent the universal challenge of stromal infiltration faced when studying this disease, and mention potential future directions [21,22].

## 2. Regulation of DNA Methylation

DNA methylation is a key epigenetic modification mechanism involving the transfer of a methyl group from S-adenosyl-L-methionine (SAM) to the 5′ carbon of cytosine, resulting in the formation of 5-methylcytosine [23]. This process primarily occurs within CpG islands—genomic regions characterized by a high frequency of cytosine-guanine dinucleotides [24]. DNA methylation plays a crucial role in regulating gene expression without altering the DNA sequence, thereby contributing to the complexity and diversity of the genome. It also serves as a stable epigenetic marker that can be inherited across multiple cell divisions and even generations, making it vital for transcriptional regulation, development, differentiation, genomic imprinting, stability, and the onset of various diseases [25,26]. Recent advances in epigenetics have shed light on the diverse mechanisms governing DNA methylation, with the majority of regulators being classified into three main categories: writers, erasers, and readers [27].

Writers include the DNA methyltransferase (DNMT) family, which catalyzes the methylation process. The DNMT family consists of DNMT1, DNMT2, DNMT3a, DNMT3b, DNMT3L, and their respective isoforms. Notably, DNMT3a and DNMT3b, often referred to as de novo methyltransferases, establish and maintain methylation patterns during development by methylating unmethylated cytosines [28]. DNMT1, on the other hand, is essential for maintaining the DNA methylome, as it preferentially targets hemimethylated DNA during replication to ensure the transmission of methylation patterns from the parent to the daughter strand, thereby functioning as a cellular memory system [29,30]. Although the classical distinction between de novo and maintenance DNA methyltransferases is well accepted in the field, there is clear evidence for functional overlap between the maintenance and the de novo methyltransferases [31].

Erasers are responsible for demethylation through either active or passive mechanisms. Active demethylation can occur through several processes: (1) enzymatic removal of the methyl group; (2) nucleotide or base excision repair, which removes methylated nucleotides; and (3) oxidation of 5-methylcytosine to 5-hydroxymethylcytosine by the TET protein family, which prevents DNMT1 recognition and maintenance [32,33].

**Figure 1:**
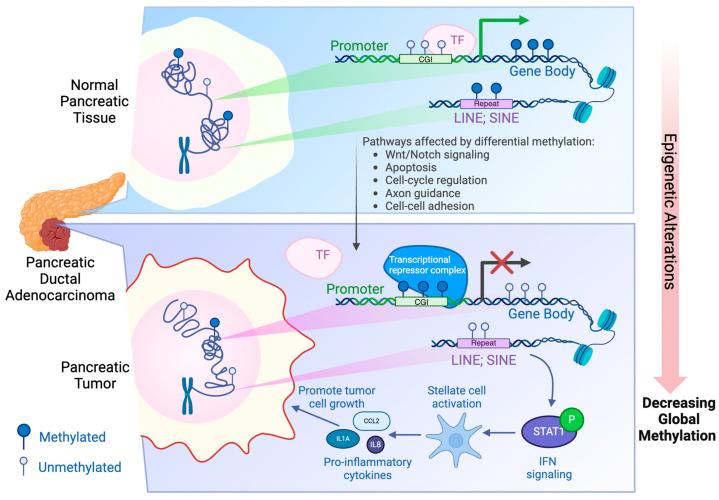
Schematic illustration of the epigenetic alterations in DNA methylation, specifically during PDAC progression. Compared to normal pancreatic tissues, PDAC has promoter CpG islands (CGI) that are hypermethylated, resulting in a closed heterochromatin state as well as gene silencing. In addition, gene bodies and intergenic regions such as repeat sequences LINE and SINE are often hypomethylated in PDAC, leading to a decreased global methylation. Hypomethylation of LINEs and SINEs activates the interferon signaling, creating a pro-inflammatory phenotype that promotes tumor cell growth and aggressiveness [21,22]. Created with BioRender.com.

The three categories of readers are the methyl-CpG-binding domain (MBD) family (MeCP2, MBD1, 2, 3, & 4), SRA domain-containing proteins, and Methyl-CpG-binding zinc fingers. Readers interpret methylation signals to regulate gene transcription, with their effects varying across different genomic regions [34]. In promoter and enhancer regions, DNA methylation is generally associated with reduced gene expression [35,36,37,38,39]. Methylation at the promoter CpGs can silence gene expression through several mechanisms: (1) physical obstruction of transcription factor binding by the methyl group in the DNA major groove; (2) failure of transcription factors to recognize methylated promoter CpG sequences; and (3) recruitment of MBD proteins to methylated DNA, which in turn attract histone deacetylases and chromatin remodelers, leading to the formation of compact, inactive heterochromatin [36,37,39] (Figure 1). A multifunctional transcription factor, CCCTC-binding factor (CTCF), acts as a reader with 11 zinc finger motifs that enable it to bind to specific DNA sequences, predominantly near the promoter regions. CTCF’s ability to recognize and bind to these sequences is sensitive to the methylation status of the DNA. When binding is inhibited by DNA methylation, interactions between promoters and cis transcription elements such as the enhancers will be blocked, silencing the gene. Although it has been suggested that CTCF plays a role in the 3D organization of the genome, it remains unclear as to whether CTCF occupancy directly contributes to overall chromatin architecture [40,41]. Conversely, DNA methylation in intergenic regions and gene bodies can be positively correlated with transcription elongation and influence gene expression in a more complex way than promoters and enhancers [42,43]. While studies have demonstrated that DNA methylation in these regions plays a role in maintaining genome stability, the regulatory mechanisms behind this process remain underexplored [44,45].

## 3. Aberrant DNA Methylation in PDAC

Dysregulation of DNA methylation is found to be closely associated with PDAC and other cancers. Numerous studies have highlighted that DNA methylation patterns deviate significantly from their normal states across various cancer types, particularly in PDAC [15,16,46] (Figure 1).

Several sodium bisulfite-based techniques have been used to detect aberrant methylation patterns in PDAC. These methods are based on the principle that sodium bisulfite treatment selectively converts unmethylated cytosine residues into uracil, while 5-methylcytosine residues remain unchanged, allowing for the differentiation between methylated and unmethylated sites [47,48]. One of the earliest techniques developed was bisulfite modification followed by methylation-specific PCR (MSP). Despite being easy to conduct, MSP has limitations: (1) it requires prior knowledge of the candidate genes, as only a limited number of CpG sites can be analyzed per primer pair [49]; (2) PCR bias leads to more efficient amplification of T-rich unmethylated sequences, compromising the accuracy of DNA methylation quantification [50]; and (3) the high concentration of bisulfite during conversion can degrade up to 90% of the DNA, leading to PCR amplification failures [51]. Microarray sequencing offers a more efficient alternative to MSP, enabling the simultaneous analysis of thousands of regions of interest within the DNA methylome. However, this method still covers only a small fraction of the methylome, often missing key regulatory and non-coding sequences.

Advancements in NGS have facilitated the development and widespread application of reduced-representation bisulfite sequencing (RRBS) and whole-genome bisulfite sequencing (WGBS). RRBS uses methylation-sensitive restriction enzymes to enrich the genomic DNA for CpG-rich regions, enabling the sequencing of methylated regions that were previously difficult to analyze with conventional bisulfite-based methods, such as repeated sequences [52,53]. WGBS, on the other hand, is the only method that provides a comprehensive profile of the DNA methylome at single base-pair resolution, identifying a significantly larger number of differentially methylated regions (DMRs) [54]. However, despite the vast number of DMRs identified through WGBS, most studies rely on association-based methods to identify candidate genes and pathways, often without establishing causality versus mere correlation in DNA methylation patterns. Further in vitro and in vivo functional studies are needed to unravel the mechanisms by which these altered DMRs drive pancreatic cancer progression and correlate with patient survival outcomes.

Using the aforementioned methods, researchers were able to identify multiple gene promoters that are aberrantly methylated within PDAC, contributing to cancer aggressiveness. Recently reported representative examples are listed in Table 1, with a more comprehensive list available in other publications [55,56]. Similar to many other types of cancers, promoter CpGs of DNA repair and tumor suppressor genes are frequently hypermethylated in PDAC, resulting in repression of these genes [31]. Conversely, oncogene promoters often exhibit hypomethylation, which drives tumor proliferation [57]. These aberrant methylation patterns are shown to be strongly associated with increased cell proliferation, invasion, metastasis, poorer prognosis, and reduced survival rates in PDAC patients (Figure 1).

Cellular pathways regulated by differential DNA methylation have also been identified. For example, Mishra et al. performed a differential methylation analysis on level-3 pancreatic cancer data from TCGA, identifying 23,688 CpG sites with differential methylation between tumor and normal samples—13,501 of these were hypermethylated, and 10,187 were hypomethylated [8,69]. They mapped 4252 genes to these differentially methylated CpGs, highlighting aberrant methylation in methylation writers, erasers, and readers, as well as homeobox genes and genes involved in pancreatic development and signaling. Subsequent pathway and gene ontology (GO) enrichment analyses revealed key enriched pathways including cancer-related pathways, MAPK signaling, calcium signaling, focal adhesion, regulation of the actin cytoskeleton, and gap junction pathways. Using Ingenuity Pathway Analysis (IPA), they identified top canonical pathways such as axonal guidance signaling, G-protein-coupled receptor signaling, hepatic fibrosis/hepatic stellate cell activation, cancer molecular mechanisms, and Sertoli cell junction pathways. Unsupervised hierarchical clustering of the data revealed three distinct patient clusters, each characterized by unique patterns of hyper- or hypomethylated CpGs, suggesting the presence of three potential pancreatic cancer subtypes within TCGA. In another study from an Indian cohort, hyper- and hypomethylation are shown to regulate 91 genes, many involved in ion transport regulation, interferon alpha/beta signaling, morphogenesis, development, and transcriptional dysregulation pathways [61]. Additionally, Bailey et al., using data from the Australian Pancreatic Cancer Genome Initiative (APGI), identified four stable PDAC subtypes: squamous, pancreatic progenitor, immunogenic, and aberrantly differentiated endocrine exocrine (ADEX) [70]. Each subtype was distinguished by distinct gene expression and DNA methylation patterns, as well as specific enriched pathways.

Collectively, these studies reveal that the DNA methylome undergoes significant rearrangement during PDAC progression, altering gene transcription patterns and pathways involved in cancer cell invasion and migration. These changes can be used to predict survival outcomes, tumor grades, and histopathological stages in PDAC patients. The strong association between methylation status and patient outcomes, particularly in genes like PD-L1 (Table 1), highlights the active role of epigenetic modifications in cancer dynamics, suggesting that DNA methylation markers could improve prognostic assessments and therapeutic targeting of pancreatic cancer.

One major challenge these above studies faced while profiling PDAC patient samples has been how to isolate or enrich neoplastic populations in given tumor samples to ensure tumor purity. PDAC is characterized by low cellularity and intense desmoplasia, resulting in a low signal-to-noise ratio that complicates the study of the molecular and cellular mechanisms of PDAC tissues. The neoplastic cellularity of the tumor samples used in the TCGA dataset ranged from 0% to 53%, with a median of 18% based on their pathology review. The tumor purity estimates from whole exome sequencing data ranged from 9% to 89%, with a median of 33% [8]. In the Bailey et al. study, among the pancreatic cancer samples analyzed, 179 samples exhibited a cellularity greater than 40%, estimated by a combination of qPure analysis of high-density SNP profiles and *KRAS* amplicon sequencing [70]. These samples were subsequently utilized for transcriptomic analyses to balance the stromal gene expression. However, a significant portion of the samples (*n* = 204) displayed lower cellularity, ranging from 12% to 40%, leading to the exclusion of these patient samples from the downstream analyses. This substantial variance in cellularity, particularly the prevalence of low-cellularity samples, underscores the urgent need for innovative methods to enhance signal-to-noise ratios or to isolate pure neoplastic populations for more accurate downstream assays and analyses.

## 4. Recent WGBS Studies on PDAC Patients

To overcome this issue, Espinet et al. recently attempted to isolate EpCAM^+^ epithelial cells and exclude cells with CD45, an immune cell marker from surgically resected primary tumor tissues using FACS [22]. They then performed WGBS with these sorted epithelial cells. While the authors showed that only a small percentage of EpCAM^−^/CD45^−^ cells had *KRAS* mutant reads in RNA-seq data, this approach could potentially neglect tumor cell heterogeneity that could account for epithelial PDAC cells that have lost EpCAM expression. In addition, it should be noted that only early-stage PDAC tumors were profiled in this study, likely due to ineligibility for surgical resection in advanced-stage PDAC patients. Nonetheless, Espinet et al. identified 56,177 DMRs with an overall predominant trend of hypomethylation in the PDAC-derived samples compared to those derived from normal pancreas. Principal component analysis (PCA) divided the tumor-derived samples into 2 distinct clusters based on DMRs. These two clusters were found to be differentially enriched in an interferon (IFN) signaling signature, as well as signatures for the two most common molecular subtypes, progenitor-like and basal-like/squamous. High expression of this interferon signature was correlated with poor prognosis and was more prevalent in tumors classified as the aggressive squamous subtype. Within the more aggressive IFN^high^ cluster, a trend of hypomethylation was found at expected partially methylated domains (PMDs), as well as in other genomic locations, notably outside of CpG islands. In particular, hypomethylation at repetitive elements (LTR/ERV, LINEs, and SINEs) was identified and associated with a higher expression of LTR/ERV and LINE-derived transcripts, the enrichment of gene signatures linked to dsRNA response, and increased IFN signaling. Their functional in vitro/in vivo experiments suggested that the interaction of PDAC cells with high IFN signaling reprograms and activates stromal cells in a cell-intrinsic process, creating a pro-tumorigenic microenvironment. Moreover, this IFN signaling was shown to be mediated by STAT1, as a JAK/STAT inhibitor impaired the tumor growth in this context. Given the close association between IFN signaling, hypomethylation at repeat elements, dsRNA response, and more aggressive cancer phenotypes, the hypomethylation of repetitive elements was suggested to activate tumoral IFN signaling, which in turn reshapes TME into a pro-tumorigenic environment, ultimately resulting in aggressive cancer phenotypes (Figure 1).

The analysis of surgically resected tumors with the aforementioned method can potentially cause sample bias, neglecting late-stage PDAC patient samples. Importantly, this issue is not limited to the study above; extensive datasets like TCGA and many other studies also exhibit similar sample biases [8]. Utilization of patient-derived organoid models can be an alternative way to complement this deficiency [71,72,73,74,75]. The ability to expand organoid cultures from a small amount of tissue, such as a fine needle aspiration (FNA) biopsy, allows us to grow cells from primary tumors, metastatic lesions, pleural effusions, and ascites of PDAC patients [71,76,77,78,79]. This method significantly enhances the representation of late-stage PDAC patients in research, providing a more comprehensive understanding of the disease’s progression. In the 3D pancreatic organoid cultures, only epithelial cells can grow and form organoids in the presence of growth factors and WNT agonists. This method effectively reduces stromal contamination in the samples, and ensures capture/expansion of all relevant epithelial cells, making it suitable for any omics studies.

To better represent various stages of PDAC patients’ epigenome, our group utilized a panel of patient-derived organoids (PDOs, *n* = 35) including human normal ductal organoids (hNs, *n* = 4), early-stage (hTs, *n* = 12), and late-stage PDAC (hTs, *n* = 19) [21]. DMR analysis between hNs and hTs identified 374 significant DMRs, with the majority (87%) being hypermethylated in PDAC organoids. These DMRs tended to be enriched in bivalent transcription start sites (TSS) and enhancer regions, suggesting that developmentally poised genes are more prone to methylation changes during PDAC progression. Subsequent GO analysis suggested that substantial epigenetic alterations occur as PDAC develops from normal pancreatic ducts, influencing the genes related to transcription, ion channels, membranes, and DNA binding. Comparative DMR analysis between early-stage and late-stage PDAC organoids further revealed 5374 DMRs, with the vast majority being hypomethylated in late-stage PDAC PDOs. These regions are significantly enriched in genes associated with cell adhesion, synapse signaling, and neurotransmitter activity, indicating a shift in cellular function and interaction as the disease progresses. We further employed DMR analysis to distinguish two major molecular subtypes of PDAC—progenitor and squamous—identifying methylation signatures that could potentially enhance PDAC subtyping and prognosis predictions [8,70,76,80,81]. The progenitor subtype exhibits a favorable prognosis characterized by the expression of transcription factors required for pancreatic endoderm cell fate determination, such as HNF4A and GATA6 [70,82,83]. Conversely, the squamous subtype, associated with a worse prognosis, shows loss of these endoderm specification TFs. Previously, DNA methylation has been implicated in the silencing of these genes [8,70]. Our study highlighted a potential role of GATA6 TF in maintaining the hypomethylated status of progenitor subtype-associated genes for their expression, likely through recruiting TET enzymes to its binding sites.

Overall, WGBS profiling of PDAC PDOs, which had not yet been explored, provides a unique opportunity to explore the DNA methylome in PDAC tumors and identify novel diagnostic markers. While it remains to be addressed whether organoid culture conditions such as growth factors and WNT signaling alter epigenomes of organoid cultures, it is obvious that PDOs provide a promising window for exploring the DNA methylome in PDAC tumors. It is important to note that there was a difference in which groups of samples had DMRs that were hypermethylated or hypomethylated across the two studies, as well as the genomic region of these DMRs. Nonetheless, both methods were proficient in segregating tumor cells from their surrounding stroma, offering new insight and direction for studying the DNA methylation landscape of PDAC.

## 5. Conclusions

The genome-wide profiling of DNA methylation of PDAC specimens in a single base- pair resolution through studies such as those conducted by Espinet et al. and Wang et al. has provided an invaluable resource for understanding the epigenetic alterations that characterize this aggressive cancer [21,22]. These studies reveal a complex landscape of differential methylation that not only distinguishes between normal and cancerous pancreatic tissues but also between different stages and molecular subtypes of the disease. The identification of specific DMRs offers potential biomarkers that could be critical for early diagnosis, prognosis, and the development of targeted therapies.

Looking to the future, the application of methylated cell-free DNA (cfDNA) circulating in the blood or methylated DNA from circulating tumor cells (CTCs) as a non-invasive diagnostic tool holds promise for the detection and management of PDAC. Methylation analysis of cfDNA provides liquid biopsy-based biomarkers, offering a practical approach for routine screening and monitoring of PDAC, which potentially enables more precise tracking of disease progression and treatment response. As the field advances, integrating findings from detailed methylome analyses with cfDNA technologies could lead to significant improvements in the specificity and sensitivity of PDAC diagnostics [84].

Moreover, the DMRs identified in these studies could serve as a critical foundation for developing cfDNA-based diagnostics. By leveraging such epigenetic signatures, researchers and clinicians could more effectively distinguish between benign conditions and malignant transformations within the pancreas, overcoming current diagnostic challenges. This approach not only promises to enhance diagnostic capabilities but also opens new avenues for personalized medicine in PDAC, where treatment strategies can be tailored based on specific epigenetic profiles. The ability to identify molecular subtypes through methylation profiling significantly enhances the potential for subtype-tailored therapies, which could improve treatment outcomes. For instance, patients identified with the classical (progenitor) subtype of PDAC might benefit specifically from tailored treatments such as FOLFIRINOX, a regimen known for its efficacy in this subgroup.

In conclusion, the identification of recurrent DMRs in PDAC underscores the functional significance of epigenetic alterations in the progression of this disease. Utilizing these DNA methylation alterations as functional biomarkers offers a promising avenue for advancing our understanding of disease progression and molecular subtyping. This approach not only enhances the precision of diagnostic and prognostic models but also opens possibilities for the development of targeted therapies tailored to specific epigenetic profiles. By integrating these epigenetic insights, we can significantly improve the strategic management of PDAC, potentially leading to better patient outcomes and a deeper comprehension of the disease’s underlying mechanisms.

## Figures and Tables

**Table 1:** Summary of gene promoters recently reported to be differentially methylated in PDAC compared to normal pancreatic tissues and their impacts on PDAC progression. ↑ denotes increase and ↓ denotes decrease.

Gene Promoter	Hypermethylated or Hypomethylated in PDAC	Affected Gene Expression Level	Molecular Function of the Gene	Functional Phenotype in PDAC	Method	Reference
PD-L1 promoter	Hypomethylated	PD-L1 ↑	Programmed cell death ligand	Aggressive clinical phenotypes ↑, immune cell infiltration ↓, expression of immune checkpoint genes ↓, overall survival ↓	Microarray	[58]
HNF4A promoter	Hypermethylated	HNF4A ↓	Controls genes that are especially important for development and function of beta cells in the pancreas; regulate cell proliferation through the Wnt/beta-Catenin Pathway	Patient survival ↓, cell growth, colony formation, and invasiveness ↑, expression decreases during early PDAC stages	Targeted bisulfite sequencing	[59]
BEND4 promoter	Hypermethylated	BEND4 ↓	BEN domain-containing protein 4 involves in non-homologous end-joining signaling by interacting with Ku80 and promotes DNA damage repair; inhibits cell growth, migration and invasion	Prognosis ↓, synthetic lethality towards ATM inhibitor	MSP	[60]
NPY and FAIM2 promoters	Hypermethylated	NPY ↓, FAIM2 ↓	NPY is involved in cell motion, invasion, and proliferation, and its hypermethylation is observed in certain cancers. FAIM2 is associated with apoptosis inhibition.	Prognosis ↓, progress to later stages ↑	Microarray	[61]
CDH13 promoter	Hypermethylated	CDH13 ↓	Cadherin, involved in cell-cell adhesion and contact inhibition of cell growth	Invasion and metastasis ↑	MSP	[62]
Cyclin D2 promoter	Hypermethylated	Cyclin D2 ↓	Involved in cell cycle regulation, cell differentiation, and neoplastic transformation	Unknown in PDAC context	MSP	[63]
SPARC promoter	Hypermethylated	SPARC ↓	A multifunctional glycoprotein, involved in cell proliferation, cell spreading, adhesion, motility, and invasion	Proliferation ↑	MSP, microarray	[64]
DKK1 promoter	Hypermethylated	DKK1 ↓	Regulates Wnt signaling by binding to the Wnt coreceptor lipoprotein-related protein-5 (LRP5)/Arrow	Proliferation ↑, migration & invasion ↑	MSP	[65]
MUC Promoter	Hypomethylated	Not tested	Mucins, bind to pathogens as part of the immune system; renewal and differentiation of the epithelium; involved in cell adhesion and signaling	Unknown in PDAC context	Microarray	[66]
KLF4 Promoter	Hypermethylated	KLF4 ↓	Zinc transcription factor, involved in cell proliferation and terminal differentiation of epithelial cells	Tumor cell differentiation ↓, proliferation ↑, patient survival ↓	MSP, targeted bisulfite sequencing	[67,68]
